# Obstetric violence in the daily routine of care and its characteristics

**DOI:** 10.1590/1518-8345.2450.3069

**Published:** 2018-11-29

**Authors:** Danúbia Mariane Barbosa Jardim, Celina Maria Modena

**Affiliations:** 1 Fundação Oswaldo Cruz, Centro de Pesquisas René Rachou, Belo Horizonte, MG, Brazil.; 2 Hospital Sofia Feldman, Belo Horizonte, MG, Brazil.

**Keywords:** Violence Against Women, Women, Obstetrics, Delivery, Exposure to Violence, Review

## Abstract

**Objective:**

to analyze the scientific production on obstetric violence by identifying and discussing its main characteristics in the routine care for the pregnant-puerperal cycle.

**Method:**

integrative literature review of 24 publications indexed in the Cumulative Index to Nursing and Allied Health Literature, Medical Literature Analysis and Retrieval System Online, SciVerse Scopus, Web of Science and the Scientific Electronic Library Online and Virtual Health Library.

**Results:**

the publications are intensified from 2015 onwards and present methodological designs of quantitative and qualitative nature. In the discussion, we first address the concept of obstetric violence and its different forms of occurrence in care. Then, interfaces of the phenomenon are presented with reflections related to the conception of gender, the different actors involved, the institutionalization, and the invisibility and trivialization of the event. Finally, strategies to combat the problem are presented through academic training, women’s awareness, proposals of social mobilization, and creation of public policies and laws.

**Conclusion:**

obstetric violence portrays a violation of human rights and a serious public health problem and is revealed in the form of negligent, reckless, omissive, discriminatory and disrespectful acts practiced by health professionals and legitimized by the symbolic relations of power that naturalize and trivialize their occurrence.

## Introduction

Birth and delivery care in Brazil has over decades been marked by significant changes brought about by the process of institutionalization that led to intense medicalization of the female body, promoting its defragmentation, depersonification and pathologization, as well as generating the abusive use of unnecessary interventions on the women and infants^1^ ^-^ ^3^.

Intersubjective and comprehensive care has gradually been replaced by complex technologies aimed at treating a defective body under the perspective that gestation is no longer understood as a physiological event of life, but rather requires excessive control and healing^1^.

In this care context, women become secondary elements in the birth scenario, subject to a controlled environment, surrounded by institutional rules and protocols that segregate them from their social and cultural context, as well as make them discredit their physiological capacity to give birth^1^ ^-^ ^2^.

Health professionals, clothed in their technical-scientific authority and backed by unequal power relationships before female users, use authority to maintain obedience to rules, disrupting human interactions and leading to the weakening of links among their patients and the crisis of confidence in the care that is provided as such approach entails the loss of the woman’s autonomy and her right to decide on matters related to her body^2^ ^-^ ^4^. These relationships are established by the imposition of unilateral authority, creating a fertile ground for the consolidation of the different forms of violence exercised during the labor and delivery care.

According to data from the World Health Organization (WHO), women are being assisted in a violent manner everywhere in world. They experience situations of mistreatment, disrespect, abuse, negligence, violation of human rights by health professionals, especially during delivery and birth^5^.

It is frequent to observe in obstetrical rooms half-naked women in the presence of strangers, or alone in unfriendly settings, in positions of total submission, open and raised legs and with genital organs exposed, and routinely separated from their children soon after birth^6^.

Reports of violence are frequent: denial of the presence of the companion of the women’s choice; lack of information about the different procedures performed during care; unnecessary cesarean sections; deprivation of the right to food and walking; routine and repetitive vaginal exams without justification; frequent use of oxytocin to accelerate labor; episiotomy without the consent of the women; and Kristeller’s maneuver. All these events can ultimately lead to permanent physical, mental and emotional damages^4^ ^,^ ^6^ ^-^ ^9^.

This scenario affects especially women of low socioeconomic status, of ethnic minorities exposed to institutional and professional power characterized by oppressive and domineering behaviors that exclude the female subjectivity as an essential feature for the construction of women-centered care and the exercise of full citizenship^5^ ^,^ ^10^ ^-^ ^11^.

Another issue raised by authors who seek to understand the phenomenon of Obstetric Violence (OV) is based on the stereotyped concept of socially widespread gender in which women, seen as the fragile sex, need to be kept under patriarchal authority (in this scenario, the physicians), who have the right to decide what is best for them, transforming the birth into a professional-centered act and subject to violent practices^4^.

Based on these observations, the guiding question of the research was: How is the phenomenon of OV characterized in the daily care during the pregnant-puerperal cycle?

The study is justified by the emerging need to know the characteristics of OV to better understand how this event occurs in the context of care and which the possible repercussions are in current obstetric practice. It is hoped that the production of this knowledge make it possible to reach different subjects - women, health professionals, managers, educational entities - who are interested in the theme, in an attempt to promote an obstetric care built free of violent acts and based on respect for sexual, reproductive and human rights. The identification and discussion of the characteristics that outline the phenomenon of OV becomes therefore important for the proposition and validation of public laws and policies favoring strategies to combat the problem and the change in the paradigms that perpetuate violent acts in the daily routine of obstetric care.

In this sense, the objective was to analyze the scientific production on OV by identifying and discussing its main characteristics in the daily routine of care for the pregnant-puerperal cycle.

## Method

The methodological strategy used to construct this text was Integrative Review of the Literature including scientific concepts derived from academic research in the search for the best scientific evidence to be applied in everyday care. This research method has the goal to gather, synthesize and analyze the existing scientific knowledge on a topic of interest of the researcher in a systematized and orderly manner, showing the evolution of the theme over the years and contributing to the deepening of research questions^(12- 13)^. In order to reach this objective, the review was based on six distinct stages, as follows: identification of the theme and selection of the research question; establishment of inclusion and exclusion criteria; identification of pre-selected and selected studies; categorization of selected studies; analysis and interpretation of results; presentation of the review^13^.

The bibliographic search was carried out based on the guiding question researched in the following virtual libraries: Scientific Electronic Library Online (SciELO) and Virtual Health Library (VHL), with access to the Specific Database of Nursing (BDENF); Spanish Bibliographical Index of Health Sciences (IBECS); Latin American and Caribbean Literature in Health Sciences (LILACS); Virtual Campus of Public Health (CVSP - Brazil); Index Psychology - Technical-scientific journals; and other databases: Cumulative Index to Nursing and Allied Health Literature (CINAHL); Medical Literature Analysis and Retrieval System Online (MEDLINE) via PubMed; SciVerse Scopus; Web of Science.

The following inclusion criteria were established: publications of a quantitative and qualitative nature, in the Portuguese, English or Spanish languages, published from 2007 to 2017 and answering the following guiding question: How is the phenomenon of OV characterized in the daily care of the pregnant-puerperal cycle? The time interval was chosen based on the desire to analyze the productions that occurred after the adoption of the Organic Law on Women’s Rights to a Life Free of Violence in 2006 in Venezuela as a landmark of the repudiation of obstetric care marked by OV. Exclusion criteria were: editorial documents (letters, comments, brief notes) and case reports.

The search strategy was initiated in the virtual libraries SciELO and VHL and replicated in the other databases. The descriptors and keywords were combined with Boolean operators: “Violência contra a mulher”, or “Violence against women”, or “Violencia contra la mujer” “(obstetric violence, or violência obstétrica) and “Obstetric delivery”, or “Delivery, obstetric” (delivery or obstetric). A total of 861 publications were initially found; their titles and abstracts were read, and the inclusion and exclusion criteria were checked, after which 801 publications were excluded.

**Figure 1:**
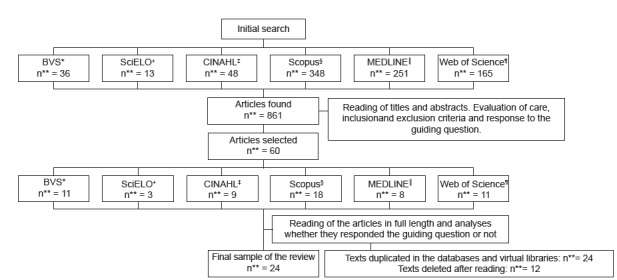
Identification, selection and inclusion of publications to compose the integrative review.

At the end, 60 publications were selected for reading in full length aiming to guarantee a greater reliability and validation of the selected material to be analyzed in this review. In this process, the texts that actually answered the question of interest and had methodological adequacy and a consistent discussion of the proposed theme were selected. After reading, the publications that presented disagreements on their suitability to compose the final sample were re-analyzed, after which they could be excluded or not. After the pre-selection and selection of the material, 24 publications were retained and composed the final sample of this review (Figure 1).

* VHL - Virtual Health Library; + SciELO - Scientific Electronic Library Online; ‡ CINAHL - Cumulative Index to Nursing and Allied Health Literature; §Scopus - Scopus bibliographic database; || MEDLINE - Medical Literature Analysis and Retrieval System Online; **n - Number.

The publications were also analyzed based on the classification proposed by Evidence-based practice, which describes seven levels of evidence: Level 1 - Evidence from systematic reviews or meta-analyses of all relevant clinical randomized controlled trials or from systematic reviews of randomized controlled trials; level 2 - evidence derived from at least one well-delineated randomized controlled trial; level 3 - evidence derived from well-delineated non-randomized clinical trials; level 4 - evidence derived from well-delineated cohort and case-control studies; level 5 - evidence derived from a systematic review of descriptive and qualitative studies; level 6 - evidence derived from a single descriptive or qualitative study; level 7 - evidence from the opinion of authorities and/or expert committee reports^14^.

After completing the methodological trajectory, the publications were thoroughly analyzed, interpreted and synthesized in a synoptic table, describing the characteristics of title, year, objectives, main results, conclusions or final recommendations.

## Results

The analysis of the data of the 24 publications included in this article revealed that 80% of them had been published in the last three years - 2015 (40%); 2016 (28%); 2017 (12%) -, reflecting the contemporaneousness of OV and the emerging need for this subject to be discussed on the world scenario. Regarding the language of publication, 36% were published in English, 28% in Spanish and 36% in Portuguese.

There was diversity as to the place of origin of the studies. It is noteworthy that 75% studies were from Latin American countries, with nine from Brazil, four from Argentina, four from Venezuela and one from Mexico; 4.2% were carried out in Europe (one study included six countries - Belgium, Iceland, Denmark, Estonia, Norway and Sweden); 8.3% in Africa (one in Kenya and one in the Republic of South Africa); and 12.5% ​​in North America (three studies in the United States).

The authors of the publications belonged to two different areas of knowledge: 75% were professionals from the Health Sciences (53% physicians, 14% nurses, 8% obstetrician nurses) and 25% from the Social Sciences and Humanities (8% lawyers, 17% anthropologists).

Regarding the distribution of designs, 32% studies had a quantitative nature, 32% had a qualitative nature and 36% were characterized as narrative-discursive. Regarding the level of evidence, 62.5% of the publications were classified as level VI (evidence derived from a single descriptive or qualitative study) and 37.5% as level VII (evidence from opinion of authorities and/or expert committee reports).

**Figure 2:**
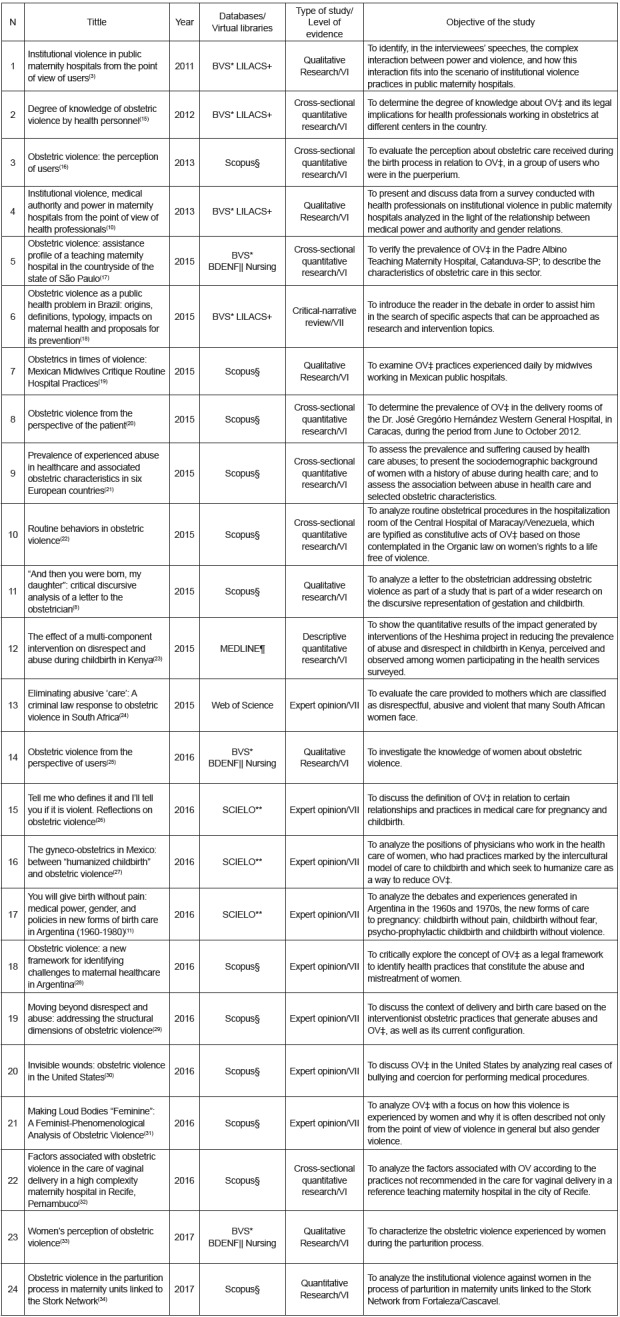
Summary of publications used in this review

For a better identification of the publications that compose this review, a summarizing table was prepared with information on: title; year of publication; reference databases and virtual libraries; classification as to the type of study; classification as to the level of evidence; and original objective of the publication (Figure 2).

* VHL - Virtual Health Library; LILACS - Latin American and Caribbean Literature in Health Sciences; ‡OV - Obstetric violence; §Scopus - Scopus bibliographic database; | BDENF - Specific Database of Nursing; ¶MEDLINE - Medical Literature Analysis and Retrieval System Online; ** SCIELO - Scientific Electronic Library Online

**Figure 3:**
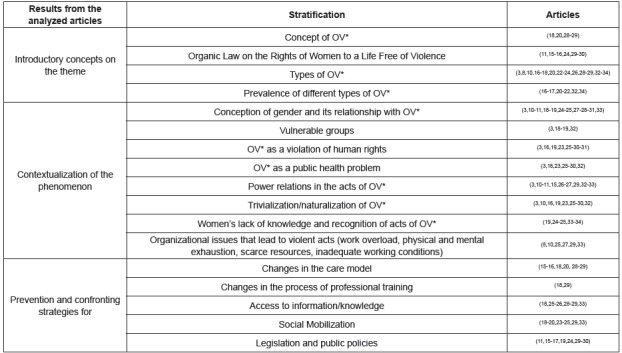
Synthesis of results found in the articles analyzed

The articles were analyzed to identify similar information shared in their results and discussions. To better understand the data, three analytical categories were prepared: Introductory concepts on the theme; Contextualization of the phenomenon and Prevention and confronting strategies. The synthesis of these elements allowed the organization of the ideas that composed the discussion, in order to characterize OV in daily care (Figure 3).

*OV - Obstetric violence.

**Figure 4:**
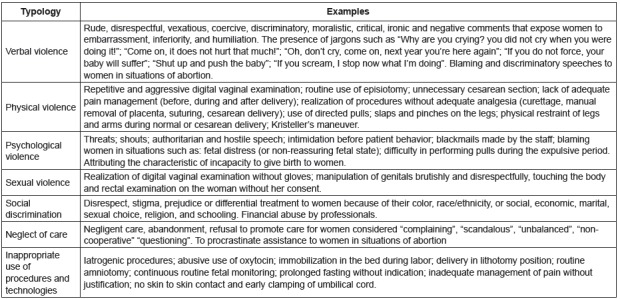
Typology and exemplification of Obstetric Violence based on the analysis of the articles included in the integrative review^3^ ^,^ ^8^ ^,^ ^10^ ^,^ ^16^ ^-^ ^18^ ^,^ ^20^ ^,^ ^22^ ^-^ ^24^ ^,^ ^26^ ^,^ ^28^ ^-^ ^29^ ^,^ ^32^ ^,^ ^34^

Different classifications for the phenomenon of OV in the context of care were identified in the articles evaluated. The information found was gathered in order to typify, illustrate the different forms of OV and show to the readers how diversified, daily and real this phenomenon is, as shown in Figure 4.

**Figure 5:**
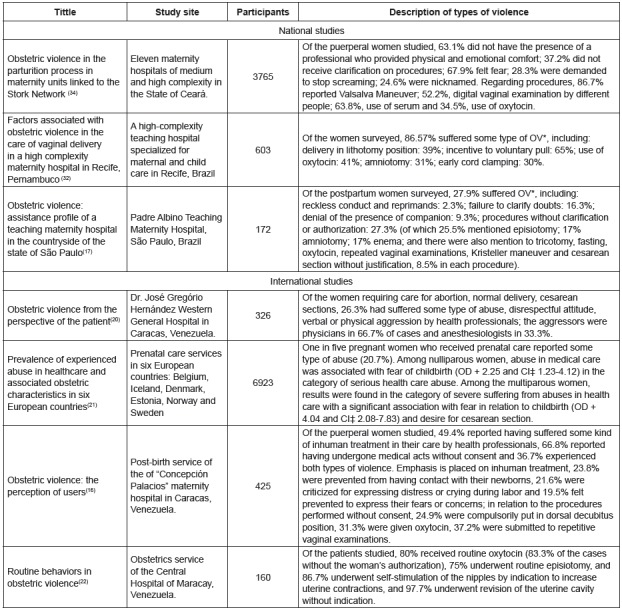
Results of the surveys included in the review, which aimed to quantify the different forms of Obstetric Violence^16^ ^-^ ^17^ ^,^ ^20^ ^-^ ^22^ ^,^ ^32^ ^,^ ^34^

The analysis of the data presented in the national and international studies that sought to quantify the different forms of OV here typified showed the findings in the Figure 5.

*OV - Obstetric Violence; +OD - Odds Ratio; ‡CI - Confidence interval

## Discussion

To understand OV, the contextualization and typification of this phenomenon are initially presented in the different care scenarios. The theoretical revision made it possible the conception of OV as a phenomenon recognized through different types of violence that may occur in the context of gestation, delivery, puerperium, as well as in situations involving assistance for cases of abortion, post-abortion and reproductive cycle^18^ ^,^ ^20^ ^,^ ^28^ ^-^ ^29^.

The main source for composition of the concept comes from the Organic Law on the Rights of Women to a Life Free of Violence, approved in November 2006 in Venezuela, which became the first country to enact a law that characterizes OV as the appropriation of the female body and reproductive processes by health professionals. This was brought up due to the inhuman treatment, abusive use of medicalization and unnecessary interventions on physiological processes, leading to loss of autonomy and freedom of choice and negatively affecting the quality of life of women^11^ ^,^ ^15^ ^-^ ^16^ ^,^ ^24^ ^,^ ^29^ ^-^ ^30^.

This context covers situations expressed in negligent, abusive, reckless, omissive, discriminatory and disrespectful acts based on relations of power and authority exercised mainly by health professionals. This may happen either in the hospital setting or in any public or private setting where acts on the female body or her sexuality can be established in a direct or indirect way, depriving women of their condition of citizens with rights before the law^3^ ^,^ ^10^ ^,^ ^20^ ^,^ ^24^ ^,^ ^28^ ^,^ ^31^ ^-^ ^32^ ^,^ ^34^. In the Statute of Violence against Women in Argentina, OV is defined as cruel, dishonorable, inhuman, humiliating, threatening treatment by health professionals, causing physical, psychological and emotional harm to assisted women^28^.

The WHO typifies forms of OV and highlights five categories that operationalize the legal definitions: 1 - routine and unnecessary interventions and medicalization (on the mother or the infant); 2 - verbal abuse, humiliation or physical aggression; 3 - lack of material and inadequate facilities; 4 - practices performed by residents and professionals without the woman’s permission after providing her comprehensive, truthful and sufficient information; 5 - discrimination on cultural, economic, religious and ethnic grounds^26^. Figure 4, previously presented in the results, exemplifies the different existing forms of OV, allowing a dimensioning of its occurrence in obstetrics and revealing its multiplicity and complexity. The recognition of the facets of this phenomenon points to the daily challenge of acting in the obstetric scenario surrounded by its existence and naturalized in actions routinely employed.

The WHO considers OV part of an entrenched institutional culture marked by the trivialization, invisibility and naturalization of the phenomenon in daily care. The described characteristics allow for the non-recognition of OV as violation of human rights and a serious global public health problem^3^ ^,^ ^16^ ^,^ ^19^ ^,^ ^23^ ^,^ ^25^ ^-^ ^30^ ^,^ ^32^.

National surveys, such as that of the Perseu Abramo Foundation, point out that one in four women in Brazil has suffered some type of OV during childbirth care and half of those who had abortions also had similar experiences. Among the forms of OV cited, 10% suffered painful vaginal examinations; 10% were denied to receive pain relief methods; 9% were treated with shouting; 9% heard cursing or were humiliated; 7% received no information about the procedures performed; 23% suffered verbal violence with prejudiced phrases^17^ ^,^ ^32^ ^,^ ^34^.

According to the results of the survey “Being born in Brazil”, 36.4% of the interviewed women (n = 23,894) received stimulant medication for childbirth; 53.5% were subjected to episiotomy; 36.1% received mechanical maneuvers to accelerate birth; 52% underwent cesarean sections without justification; 55.7% were kept restricted to the bed; 74.8% were subjected to fasting and 39.1% underwent amniotomy^17^. The findings of the previous research converge with other data found in this review regarding the quantification of the different forms of OV presented in Figure 5 in the results of the present article.

Reflecting on OV, its subjects, actors and possible justifications, different views are observed in everyday care with a highlight on fundamental discussions for understanding, appropriation, social mobilization and categories in defense of women victims of this event.

Possible explanations for its occurrence are encouraged by the authors, starting from an initial analysis of the existence of a group of women more vulnerable to the different forms of OV. This group is made up of black women, or those belonging to ethnic minorities, adolescents, poor, with low school education, drug users, homeless women, women without prenatal care, and without companion at the time of care^3^ ^,^ ^18^ ^-^ ^19^ ^,^ ^32^.

Besides the establishment of a more exposed group, the authors mention a deep relationship between the representation of gender ideology and the occurrence of OV. The culturally consolidated image of women as reproductive, submissive, and physically and morally inferior opens a precedent for the domination, control, abuses and coercion of their bodies and their sexuality, intertwined by discriminatory issues^(3,10-11,18- 19,24-25,28-31,33)^. In this conception of gender, women are objectified, labeled naturally as reproductive bodies. Their subjectivity is annulled and they are deprived of any right of choice^3^ ^,^ ^31^ ^,^ ^33^.

OV is a feminist question, the result of patriarchal oppression that leads to the undervaluation, oppression and objectification of the female body limiting the power and ways of expression of the women. Contrary to the masculine thought of fragilization, the female body is strong, active, creative, capable of supporting situations such as labor and delivery and, for this reason, it needs domestication and control to reduce it to the condition of object, “deactivated”, alienated, omissive, thus the aim of violation^31^. Women, in this scenario, are deprived of their identity, fragmented, leaving their totality and becoming only wombs, a shelter for the fetus, a machine to make babies or simply the “mother” ^(3,15,19,28 )^.

Violent acts are practiced by health professionals - mostly doctors - based on their technical and scientific knowledge, by hierarchical and unequal power and authority relations, in a hegemonic and patriarchal biomedical model that segregates and illegitimates the power of females over their bodies, making them passive and disciplined^3^ ^,^ ^10^ ^,^ ^15^ ^,^ ^26^ ^,^ ^29^ ^,^ ^32^ ^-^ ^33^.

The relationship of trust between women and health professionals is torn, generating fragilization of the existing links and loss of human singularity and subjectivity. Before the symbolic legitimacy that “knowledge-power” imposes on physicians, however, the woman is subject to agree with the wills of the professionals, becoming dependent, subordinate and hostage of this violent cycle, fueled by fear and insecurity about obstetric processes^3^ ^,^ ^10^ ^-^ ^11^ ^,^ ^26^.

Another important reflection pointed out by some authors is based on the paradox between OV by female health professionals, at times identified as torturers and more violent than their male peers in the obstetric practice. There is a denial of the phenomenon of feminization of gynecological-obstetric care associated with the growing problem of OV and gender issues. The dichotomy in this process is also emphasized because they are executors and potentially victims when they need assistance in some obstetric demand^27^.

The health professional, in turn, has difficulty identifying himself as the author of OV in its different forms, transposing the practice into natural, justifiable and necessary acts that would be performed for the “good” of the patients and their babies, thus legitimizing their actions^10^ ^,^ ^26^ ^-^ ^27^ ^,^ ^33^. This way of acting de-characterizes violence in its ethical-moral aspect and creates desirable ways to accept and qualify violent acts in obstetric care. The banality of OV, discreetly naturalized in behaviors considered as “jokes” by health professionals, is even expected by the patients, who, socially, spread this reality to other women as a normal part of daily life^10^.

Another explanation commonly given by professionals in the attempt to “justify” the violent scenario of obstetric care is based on elements such as work overload, scarce human resources, physical and mental exhaustion of professionals, precariousness of the conditions for care provision, and lack of adequate infrastructure in institutions. These problems altogether generate stressful, disqualified environments favorable to the occurrence of the different types of OV, culminating in the lack of commitment of health professionals, who also feel violated by inadequate working conditions^8^ ^,^ ^10^ ^,^ ^27^ ^,^ ^29^. Moved by a feeling of impunity and passivity, health professionals perpetuate violent practices during obstetric care, replacing ethical relationships with inhuman, highly technological and invasive care^10^.

Another important counterpoint for the persistence of violent acts in obstetric care is the lack of knowledge of women about their sexual and reproductive rights. In reality, women are unable to realize whether or not they suffered violent acts because they trust the caregivers, and also because of the very physical and emotional fragility that obstetric processes entail. They end up accepting procedures without any questioning, they do not express their desires, their doubts and they suffer in silence without even knowing they were violated^19^ ^,^ ^24^ ^-^ ^25^ ^,^ ^33^ ^-^ ^34^. This passivity allows the authoritarian imposition of derogatory norms and moral values by health professionals who, once again, judge what is best for patients, putting them in a situation of impotence^25^ ^,^ ^33^.

Some strategies for the prevention and confrontation of OV are proposed in the texts analyzed in this review. Changes that cover multiple dimensions are discussed, such as the the obstetric care model in force in the world, the awareness of women and of the general population about the theme and their rights, and the promotion of research on topics related to OV, seeking to elucidate questions that have not been answered in the existing studies^18^ ^,^ ^29^.

Some authors emphasize the importance of profound changes in the training model of human resources in the health area, both in undergraduate and postgraduate courses. Themes such as the sexual and reproductive rights of women, gender relations, code of ethics, physiological assistance to labor and delivery, humanization of obstetrical care, teaching of evidence-based practice should be part of the academic routine of future professionals, prompting reflections on the current context and on what changes are necessary for the construction of a respectful, human and comprehensive assistance^18^ ^,^ ^29^.

Another important point highlighted by the authors refers to the investments required for the training of obstetrical nurses and obstetricians who assist of physiological deliveries and positively affect the reduction of iatrogenic procedures, the promotion of humanized labor and the reduction of unnecessary cesarean sections^18^.

With regard to interventions for women, the authors emphasize the need to provide information on issues involving OV and access to the evidence-based and unbiased information on obstetric interventions, promoting the empowerment of women as subjects of law and their autonomy in the care provided to them^18^ ^,^ ^25^ ^-^ ^26^ ^,^ ^28^ ^-^ ^29^ ^,^ ^33^.

Fundamental rights in obstetric care should be guaranteed and grounded on demedicalization of birth and evidence-based practice. Issues such as the presence of a companion, the possibility of birth in a vertical position, compliance with the woman’s birth plan, free and informed consent before performing medical procedures (such as episiotomy, cesarean section), and the reasonable and adequate use of technologies should be respected^15^ ^-^ ^16^ ^,^ ^18^ ^-^ ^20^ ^,^ ^28^.

In actions aimed at raising the awareness of the general population about the issue of OV, it is fundamental to give visibility to the problem with the creation of channels for denunciation and accountability of the different actors involved - institutions, managers, health professionals, Public Prosecutors, and Public Defenders. In recent years, initiatives related to women’s movements, governmental and non-governmental organizations and civil society have contributed to the wide discussion of this phenomenon and the creation of strategies for denouncing, confronting and punishing the responsible for such acts, stressing the need that these groups become get involved in the decisions that need to be taken in the struggle to end the various forms of violence^18^ ^-^ ^20^ ^,^ ^23^ ^-^ ^25^ ^,^ ^29^ ^,^ ^33^.

The creation of laws, ordinances and public policies to protect women against OV, acknowledging their right to a care free of violence and the autonomy over their bodies must be pursued. It is necessary to struggle for the judicial entities to consider the VO as an offense with attribution of penalties, which may vary from payment of fines, disciplinary proceedings to convictions of imprisonment through the judgment of the acts committed by the perpetrators^(11,15-16,19,24 , 29-30)^. The confrontation of OV depends on the dissemination of information to civil society, women, social movements, health professionals, institutions about the existence of these regulations and the legal repercussions of the practice of violence in the obstetric scenario^(15,20,23, 29,33)^.

It is not enough, however, to punish perpetrators; it is necessary to promote prevention actions and, in some cases, to repair existing situations in search of a respectful, dignified obstetrical care that promote change, as well as the sharing of responsibilities among all those involved in the process, namely, health professionals and service managers^17^ ^,^ ^30^.

To conclude the discussion proposed in this review, we highlight some advances in knowledge such as the listing of forms of OV that allows the identification of its occurrence in obstetric care and reveals to health professionals the challenge of offering women care free of violence. The proposed reflections sought to clarify the main explanations for the subsistence of OV, allowing the proposal of new debates on raising issues such as strategies to enhance the awareness of Institutions, care professionals and class entities on the theme.

It is, therefore, necessary to advance the discussion about the forms of combating OV at the national level and the possible strategies for implementing these actions in the different obstetrical services. The difficulty in obtaining data about the occurrence and characteristics of OV in services of the supplementary network made it impossible to make a more complete analysis of the theme in this scenario and suggests the importance of expanding the studies contemplating the women assisted in these services.

## Conclusion

The synthesis of the findings of the studies allowed the identification of characteristics of OV as an obvious event expressed as negligent, reckless, omissive, discriminatory and disrespectful acts practiced by health professionals and legitimized by the symbolic relations of power and the technical-scientific knowledge that naturalize and trivialize its occurrence in the obstetric scenario. Thus, VO depicts a violation of human rights and constitutes a serious public health problem.

It should be stressed that the proposal of strategies to prevent and combat this event involves academic training, women’s awareness, social mobilization, and the creation of laws and public policies in a joint challenge to guarantee the provision of obstetric care free of violence and the respect of sexual and reproductive rights.

## References

[1] Torres JA, Santos I, Vargens OMC (2008). Constructing a care technology conception in obstetric nursing: a
sociopoetic study. Texto Contexto Enferm.

[2] Sena LM, Tesser CD (2017). Obstetric violence in Brazil and cyberactivism of mothers: report
of two experiences. Interface Comun Saúde Educ.

[3] Aguiar JM, d’Oliveira AFPL (2011). Institutional violence in public maternity hospitals: the women’s
view. Interface Comun Saúde Educ.

[4] Bellón Sánchez S (2015). Obstetric violence from the contributions of feminist criticism
and biopolitics. Dilemata Int J Appl Ethics.

[5] World Health Organization (2014). The prevention and elimination of disrespect and abuse during
facility-nbased chidlbirth.

[6] Fernández Guillén F (2015). What is obstetric violence? Some social, ethical and legal
aspects. Dilemata Int J Appl Ethics.

[7] Pérez D’Gregorio R (2010). Obstetric violence: a new legal term introduced in
Venezuela. Int J Gynaecol Obstet.

[8] Regis JFS, Resende VM (2015). “Then you delivered my daughter”: critical discourse analysis of
a letter to the obstetrician. DELTA.

[9] Gómez Pérez BA, Oliveira EV, Lago MS (2015). Perceptions of postpartum during labor and delivery: integrative
review. Rev Enferm Contemp.

[10] Aguiar JM, d’Oliveira AFPL, Schraiber LB (2013). Institutional violence, medical authority, and power relations in
maternity hospitals from the perspective of health workers. Cad Saúde Pública.

[11] Felitti K (2011). Without pain you will bring forth children: medical power,
gender, and politics in new forms of assisted childbirth in Argentina
(1960-1980). Hist Cienc Saude Manguinhos.

[12] Mendes KDS, Silveira RCCP, Galvão CM (2008). Integrative literature review: a research method to incorporate
evidence in health care and nursing. Texto & Contexto Enferm.

[13] Souza MT, Silva MD, Carvalho R (2010). Integrative review: what is it? How to do it?. Einstein (São Paulo).

[14] Melnyk BM, Fineout-Overholt E (2011). Evidence-based practice in nursing and healthcare: a guide to best
practice [Internet].

[15] Faneite J, Feo A, Toro Merlo J (2012). Grado de conocimiento de violencia obstétrica por el personal de
salud. Rev Obstet Ginecol Venezuela.

[16] Terán P, González Blanco M, Ramos D, Castellanos C (2013). Violencia obstétrica: percepción de las usuarias. Rev Obstet Ginecol Venezuela.

[17] Biscegli TS, Grio JM, Melles LC, Ribeiro SRMI, Gonsaga RAT (2015). Obstetrical violence: profile assistance of a state of São Paulo
interior maternity school. Cuid Arte Enferm.

[18] Diniz SG, Salgado HO, Andrezzo HFA, Carvalho PGC, Carvalho PCA, Aguiar CA (2015). Abuse and disrespect in childbirth care as a public health issue
in Brazil: origins, definitions, impacts on maternal health, and proposals
for its prevention. J Hum Growth Dev.

[19] Zacher Dixon L (2015). Obstetrics in a time of violence: Mexican midwives critique
routine hospital practices. Med Anthropol Q.

[20] Pereira C, Toro J, Domínguez A (2015). Violencia obstétrica desde la perspectiva de la
paciente. Rev Obstet Ginecol Venezuela.

[21] Lukasse M, Schroll AM, Karro H, Schei B, Steingrimsdottir T, Van Parys AS (2015). Prevalence of experienced abuse in healthcare and associated
obstetric characteristics in six European countries. Acta Obstet Gynecol Scand.

[22] Camacaro M, Ramírez M, Lanza L, Herrera M (2015). Routine behaviors in birth care that constitute obstetrical
violence. Utopía y Praxis Latinoamericana.

[23] Abuya T, Ndwiga C, Ritter J, Kanya L, Bellows B, Binkin N (2015). The effect of a multi-component intervention on disrespect and
abuse during childbirth in Kenya. BMC Pregnancy Childbirth.

[24] Pickles C (2015). Eliminating abusive ‘care’: A criminal law response to obstetric
violence in South Africa. SA Crime Quart.

[25] Silva RLV, Lucena KDT, Deininger LSC, Martins VS, Monteiro ACC, Moura RMA (2016). Obstetrical violence under the look of users. Rev Enferm UFPE On Line.

[26] Castrillo B (2016). Tell me by whom is defined and i’ll tell if it is violent: a
reflection on obstetric violence. Sex Salud Soc. (Rio J.).

[27] Pozzio MR (2016). The gynecology obstetrics in México: between “humanized
childbirth” and obstetric violence. Rev Estud Fem.

[28] Vacaflor CH (2016). Obstetric violence: a new framework for identifying challenges to
maternal healthcare in Argentina. Reprod Health Matters.

[29] Sadler M, Santos MJ, Ruiz-Berdún D, Rojas GL, Skoko E, Gillen P (2016). Moving beyond disrespect and abuse: addressing the structural
dimensions of obstetric violence. Reprod Health Matters.

[30] Diaz-Tello F (2016). Invisible wounds: obstetric violence in the United
States. Reprod Health Matters.

[31] Shabot SC (2016). Making loud bodies “feminine”: a feminist-phenomenological
analysis of obstetric violence. Hum Stud.

[32] Andrade PON, Silva JQP, Diniz CMM, Caminha MFC (2016). Factors associated with obstetric abuse in vaginal birth care at
a high-complexity maternity unit in Recife, Pernambuco. Rev Bras Saúde Mater Infant.

[33] Oliveira TR, Costa REOL, Monte NL, Veras JMMF, Sá MIMR (2017). Women’s perception on obstetric violence. Rev Enferm UFPE On Line.

[34] Rodrigues FA, Lira SVG, Magalhães PH, Freitas ALV, Mitros VMS, Almeida PC (2017). Violence obstetric in the parturition process in maternities
linked to the Stork Network. Reprod Clim.

